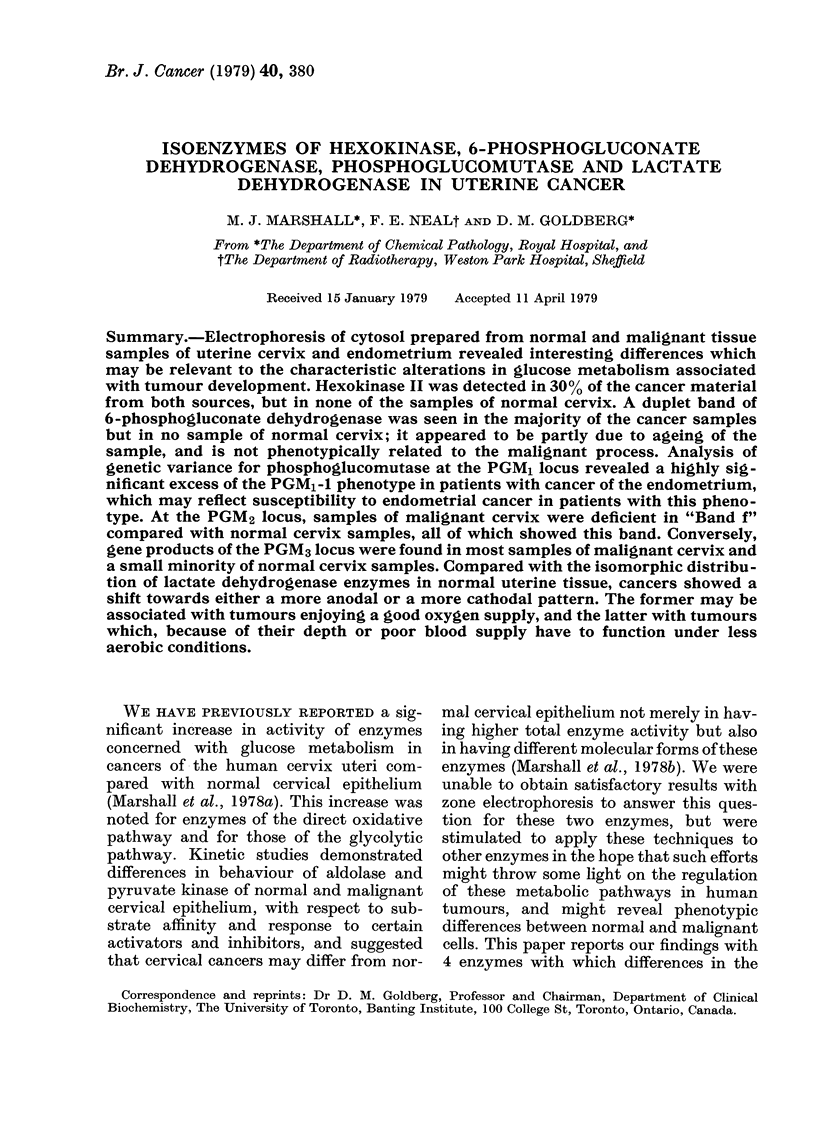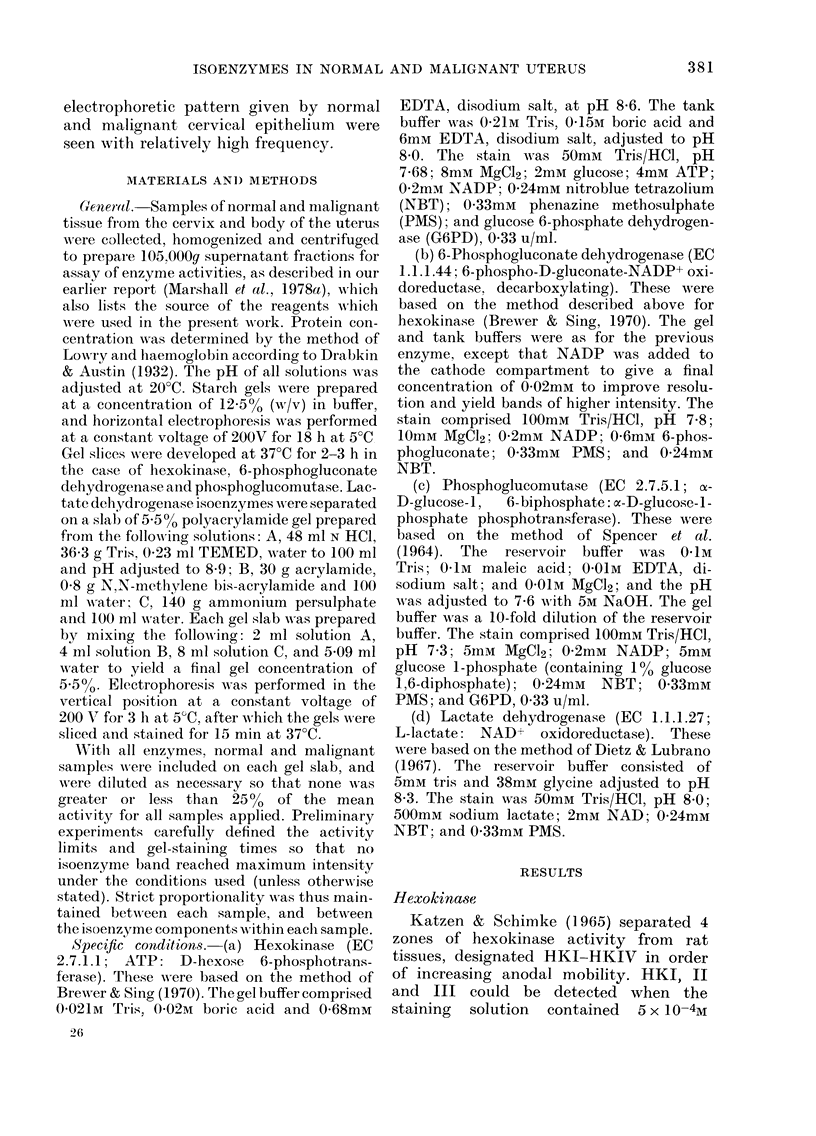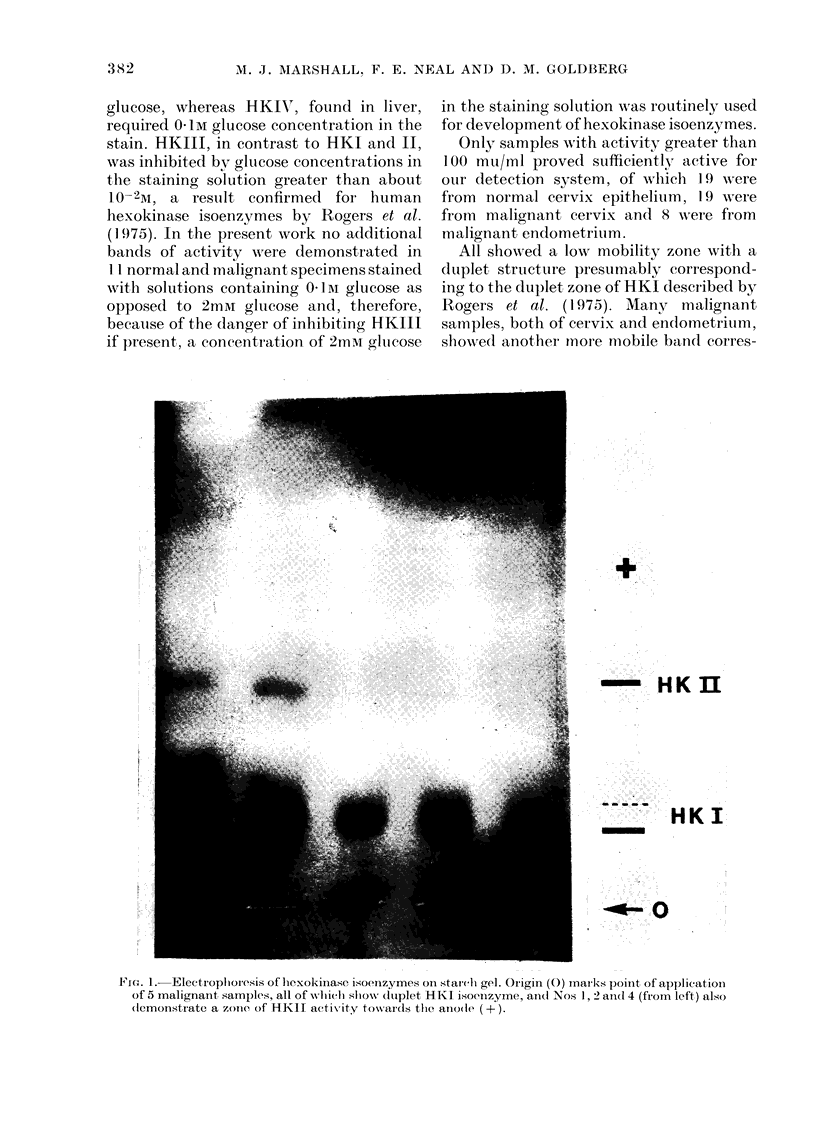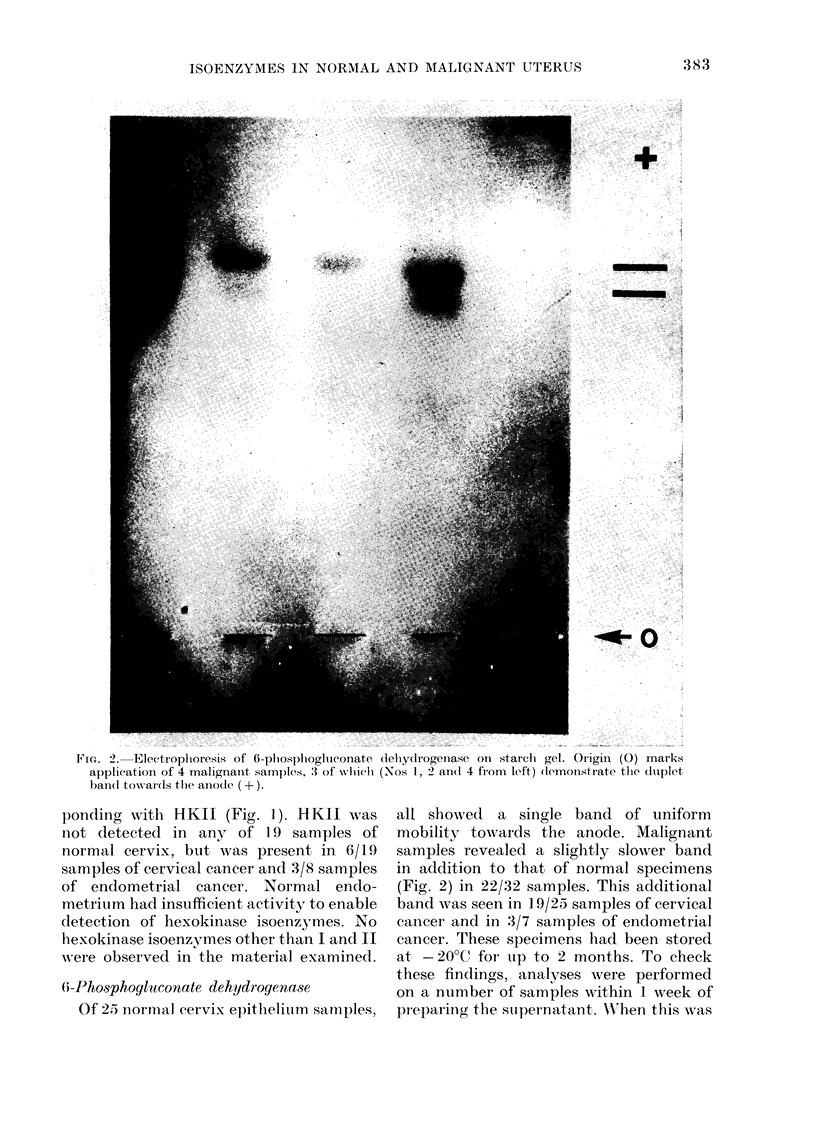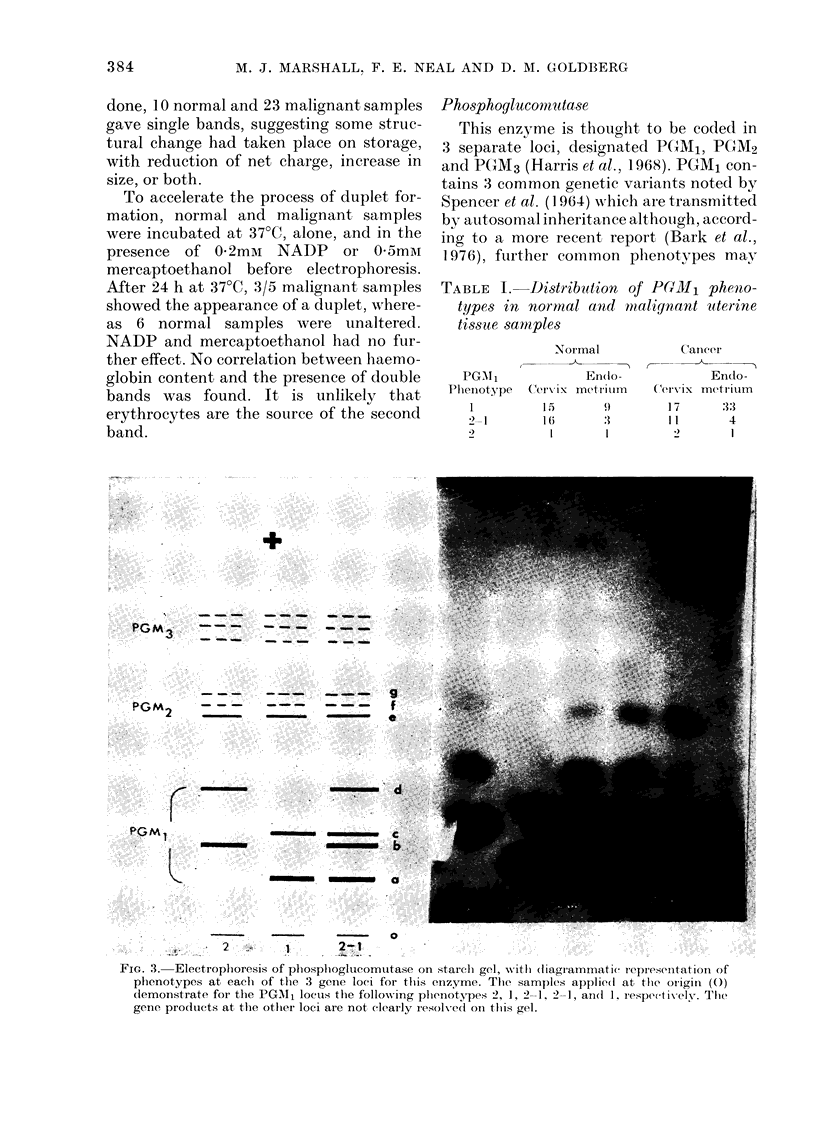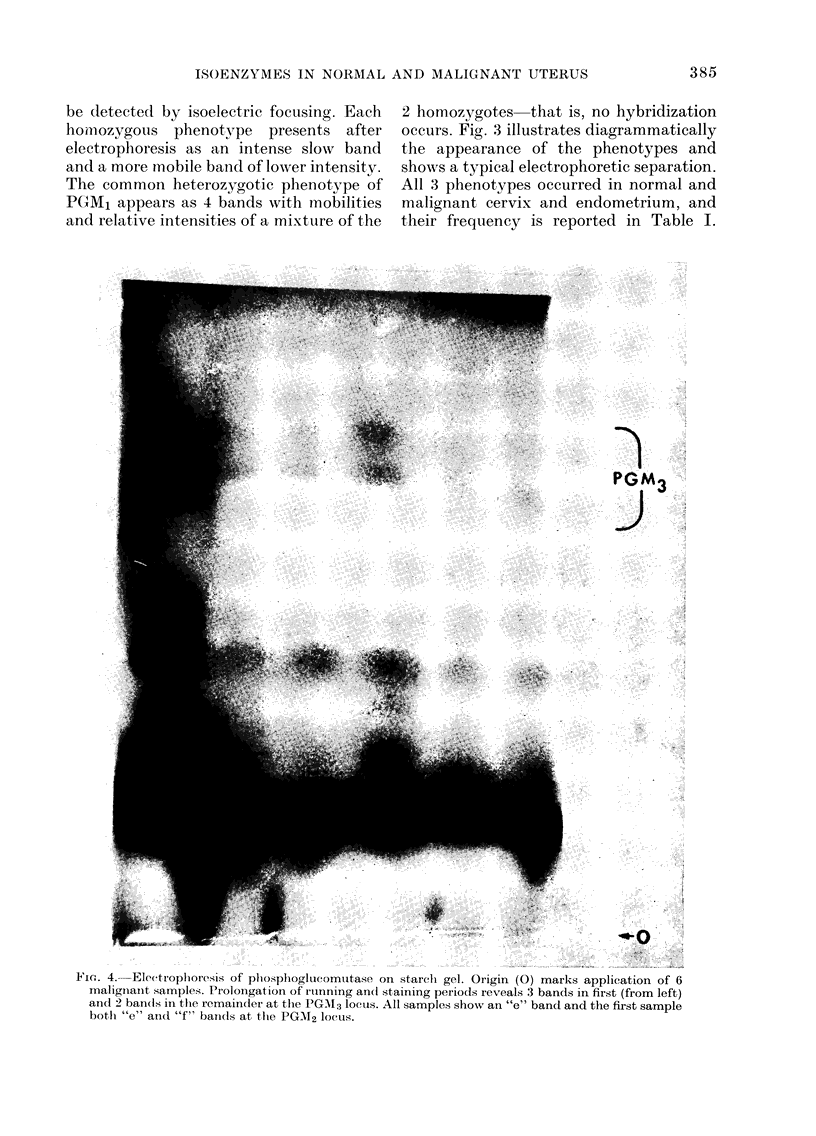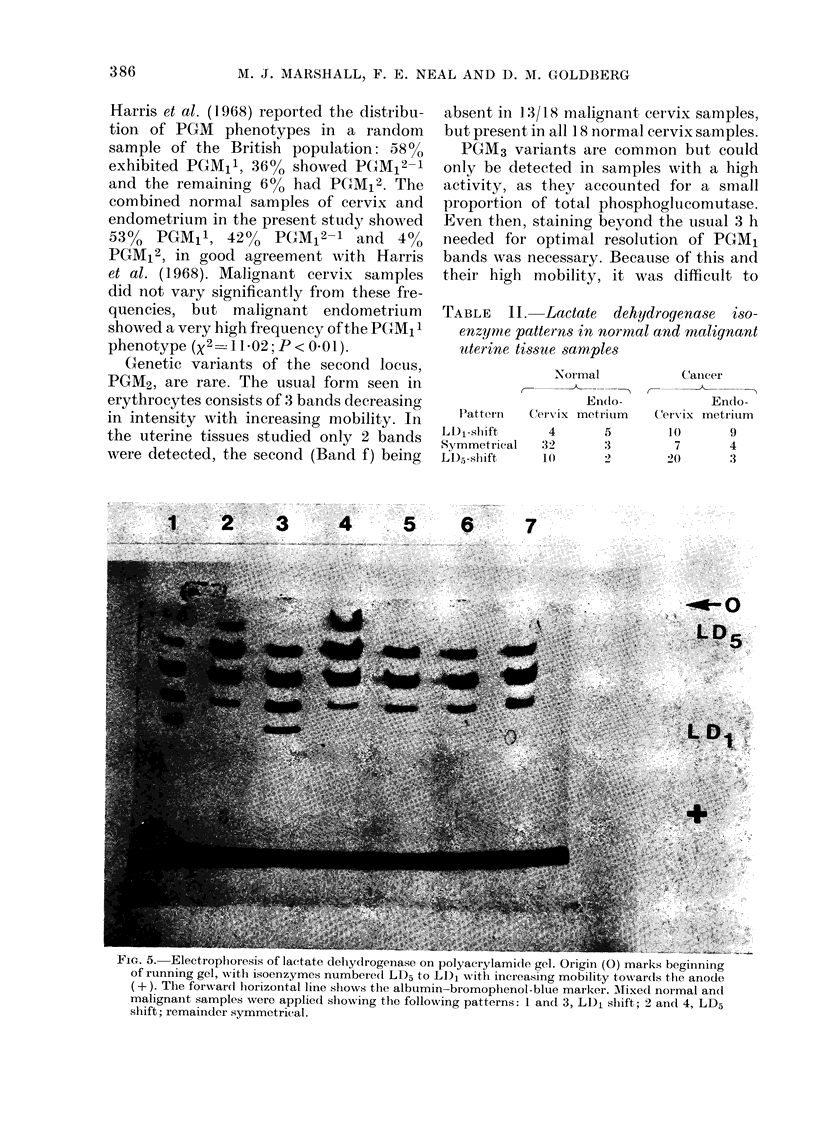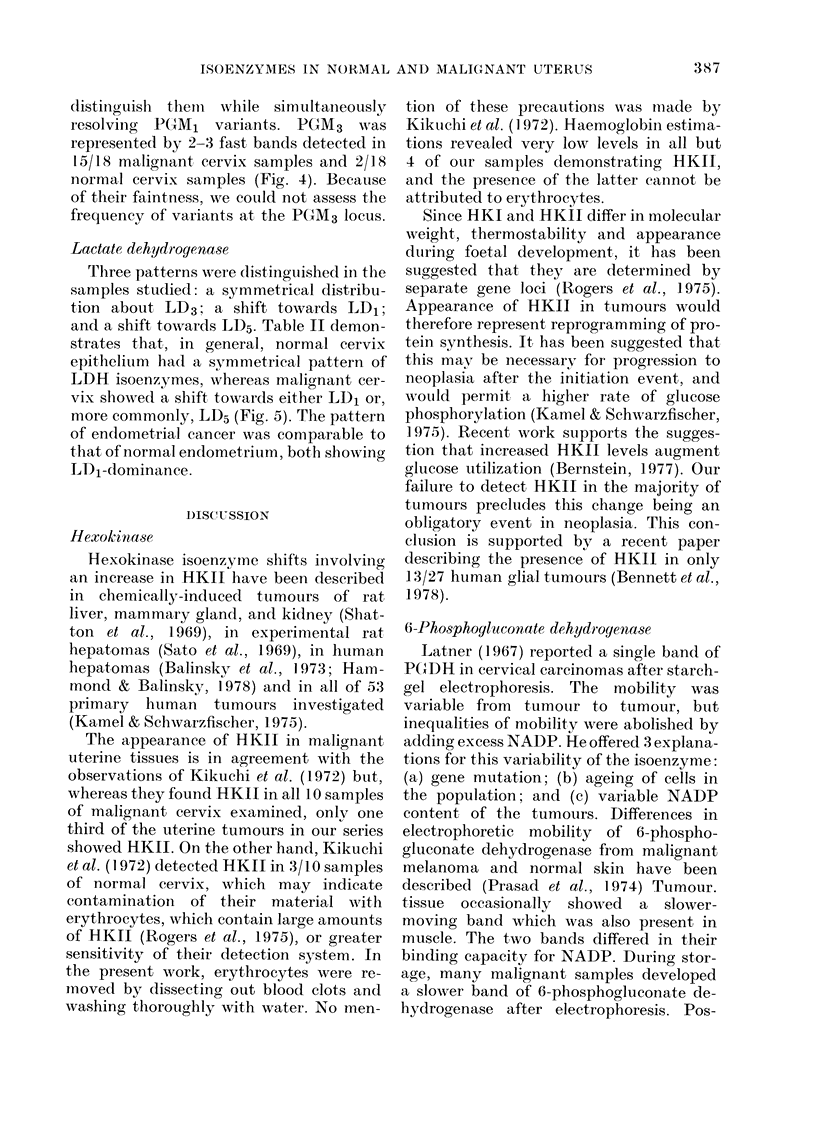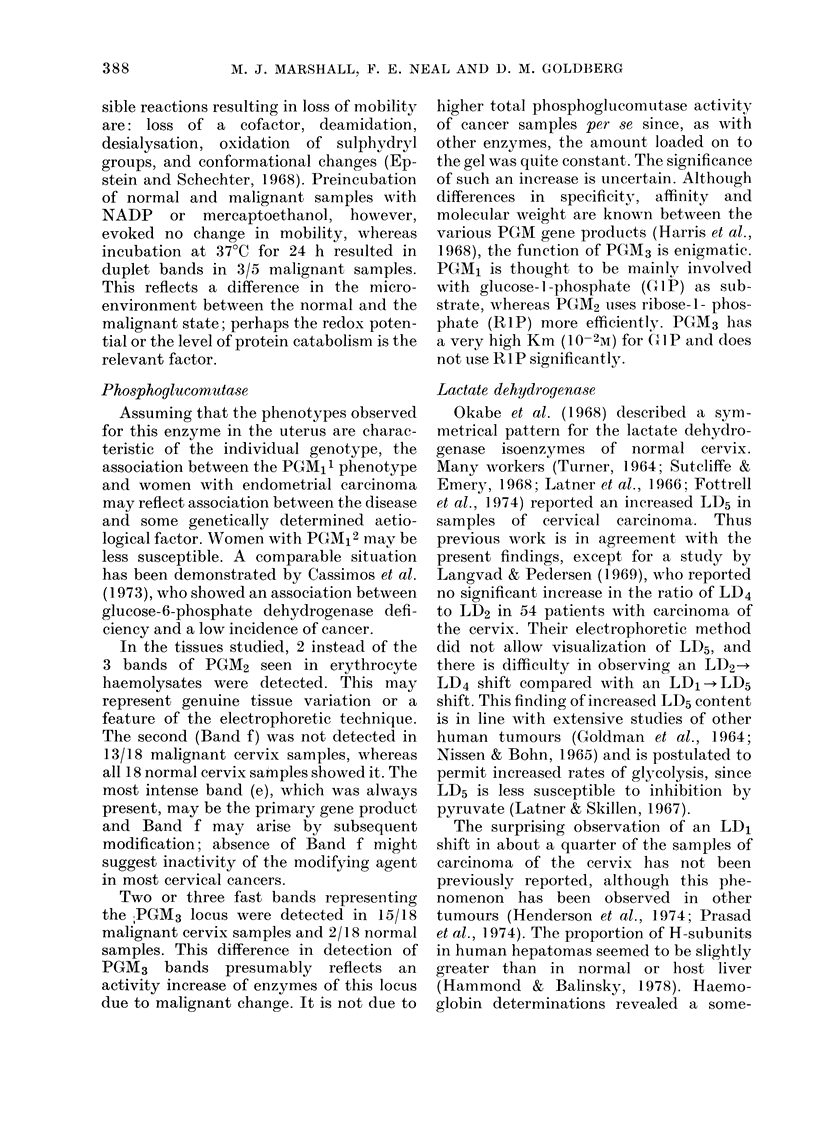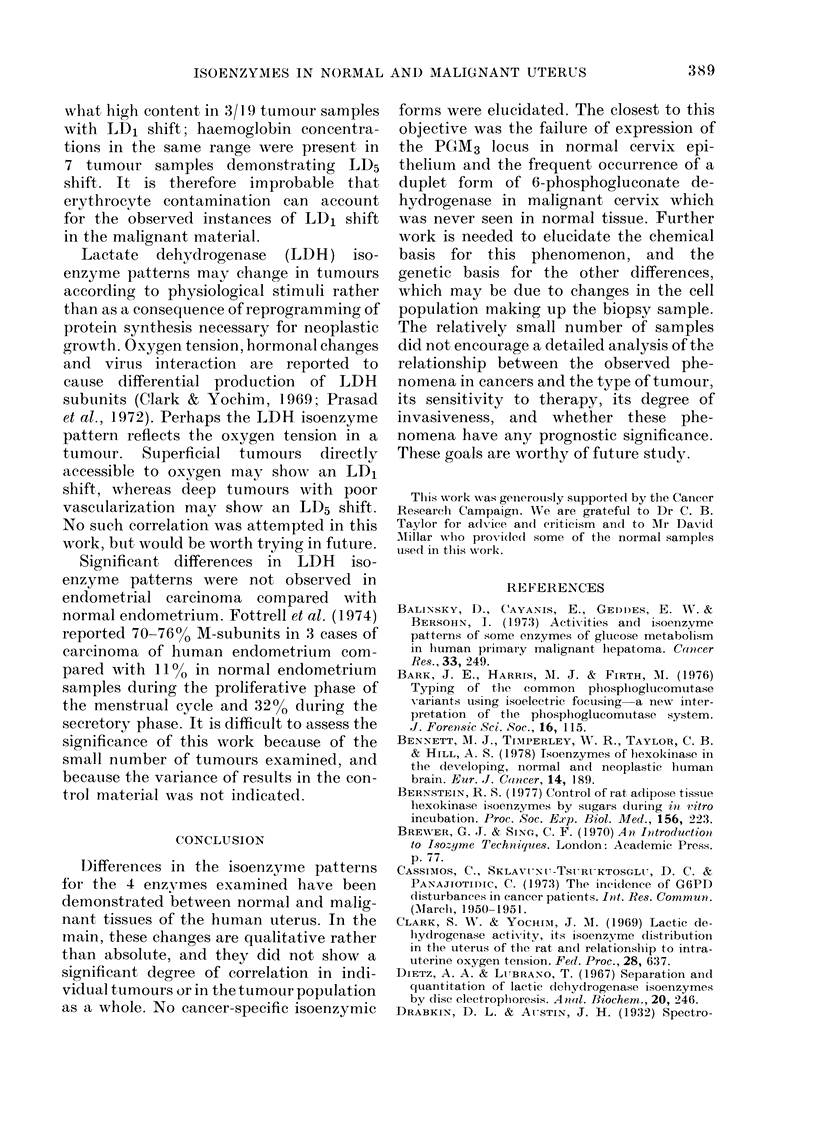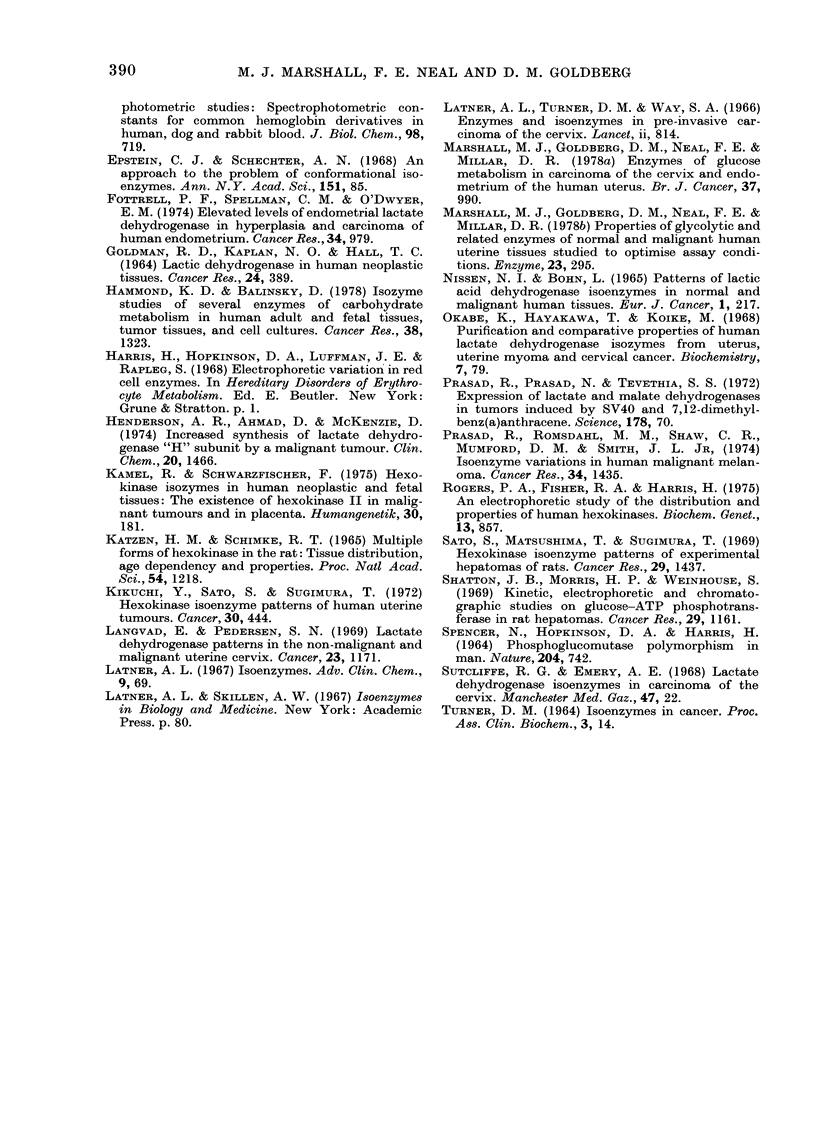# Isoenzymes of hexokinase, 6-phosphogluconate dehydrogenase, phosphoglucomutase and lactate dehydrogenase in uterine cancer.

**DOI:** 10.1038/bjc.1979.192

**Published:** 1979-09

**Authors:** M. J. Marshall, F. E. Neal, D. M. Goldberg

## Abstract

**Images:**


*Br. J. Cancer* (1979) **40**, 380

### ISOENZYMES OF HEXOKINASE, 6-PHOSPHOGLUCONATE DEHYDROGENASE, PHOSPHOGLUCOMUTASE AND LACTATE DEHYDROGENASE IN UTERINE CANCER

M. J. MARSHALL*, F. E. NEAL† AND D. M. GOLDBERG*

*From *The Department of Chemical Pathology, Royal Hospital, and †The Department of Radiotherapy, Weston Park Hospital, Sheffield*

Received 15 January 1979 Accepted 11 April 1979

**Summary.—Electrophoresis of cytosol prepared from normal and malignant tissue samples of uterine cervix and endometrium revealed interesting differences which may be relevant to the characteristic alterations in glucose metabolism associated with tumour development. Hexokinase II was detected in 30% of the cancer material from both sources, but in none of the samples of normal cervix. A duplet band of 6-phosphogluconate dehydrogenase was seen in the majority of the cancer samples but in no sample of normal cervix; it appeared to be partly due to ageing of the sample, and is not phenotypically related to the malignant process. Analysis of genetic variance for phosphoglucomutase at the $PGM_1$ locus revealed a highly significant excess of the $PGM_1$-1 phenotype in patients with cancer of the endometrium, which may reflect susceptibility to endometrial cancer in patients with this phenotype. At the $PGM_2$ locus, samples of malignant cervix were deficient in "Band f" compared with normal cervix samples, all of which showed this band. Conversely, gene products of the $PGM_3$ locus were found in most samples of malignant cervix and a small minority of normal cervix samples. Compared with the isomorphic distribution of lactate dehydrogenase enzymes in normal uterine tissue, cancers showed a shift towards either a more anodal or a more cathodal pattern. The former may be associated with tumours enjoying a good oxygen supply, and the latter with tumours which, because of their depth or poor blood supply have to function under less aerobic conditions.**

We HAVE PREVIOUSLY REPORTED a significant increase in activity of enzymes concerned with glucose metabolism in cancers of the human cervix uteri compared with normal cervical epithelium (Marshall *et al.*, 1978*a*). This increase was noted for enzymes of the direct oxidative pathway and for those of the glycolytic pathway. Kinetic studies demonstrated differences in behaviour of aldolase and pyruvate kinase of normal and malignant cervical epithelium, with respect to substrate affinity and response to certain activators and inhibitors, and suggested that cervical cancers may differ from normal cervical epithelium not merely in having higher total enzyme activity but also in having different molecular forms of these enzymes (Marshall *et al.*, 1978*b*). We were unable to obtain satisfactory results with zone electrophoresis to answer this question for these two enzymes, but were stimulated to apply these techniques to other enzymes in the hope that such efforts might throw some light on the regulation of these metabolic pathways in human tumours, and might reveal phenotypic differences between normal and malignant cells. This paper reports our findings with 4 enzymes with which differences in the

Correspondence and reprints: Dr D. M. Goldberg, Professor and Chairman, Department of Clinical Biochemistry, The University of Toronto, Banting Institute, 100 College St, Toronto, Ontario, Canada.

electrophoretic pattern given by normal and malignant cervical epithelium were seen with relatively high frequency.

### MATERIALS AND METHODS

*General.*—Samples of normal and malignant tissue from the cervix and body of the uterus were collected, homogenized and centrifuged to prepare 105,000*g* supernatant fractions for assay of enzyme activities, as described in our earlier report (Marshall *et al.*, 1978*a*), which also lists the source of the reagents which were used in the present work. Protein concentration was determined by the method of Lowry and haemoglobin according to Drabkin & Austin (1932). The pH of all solutions was adjusted at 20°C. Starch gels were prepared at a concentration of 12·5% (w/v) in buffer, and horizontal electrophoresis was performed at a constant voltage of 200V for 18 h at 5°C. Gel slices were developed at 37°C for 2–3 h in the case of hexokinase, 6-phosphogluconate dehydrogenase and phosphoglucomutase. Lactate dehydrogenase isoenzymes were separated on a slab of 5·5% polyacrylamide gel prepared from the following solutions: A, 48 ml N HCl, 36·3 g Tris, 0·23 ml TEMED, water to 100 ml and pH adjusted to 8·9; B, 30 g acrylamide, 0·8 g N,N-methylene bis-acrylamide and 100 ml water; C, 140 g ammonium persulphate and 100 ml water. Each gel slab was prepared by mixing the following: 2 ml solution A, 4 ml solution B, 8 ml solution C, and 5·09 ml water to yield a final gel concentration of 5·5%. Electrophoresis was performed in the vertical position at a constant voltage of 200 V for 3 h at 5°C, after which the gels were sliced and stained for 15 min at 37°C.

With all enzymes, normal and malignant samples were included on each gel slab, and were diluted as necessary so that none was greater or less than 25% of the mean activity for all samples applied. Preliminary experiments carefully defined the activity limits and gel-staining times so that no isoenzyme band reached maximum intensity under the conditions used (unless otherwise stated). Strict proportionality was thus maintained between each sample, and between the isoenzyme components within each sample.

*Specific conditions.*—(a) Hexokinase (EC 2.7.1.1; ATP: D-hexose 6-phosphotransferase). These were based on the method of Brewer & Sing (1970). The gel buffer comprised 0·021M Tris, 0·02M boric acid and 0·68mM EDTA, disodium salt, at pH 8·6. The tank buffer was 0·21M Tris, 0·15M boric acid and 6mM EDTA, disodium salt, adjusted to pH 8·0. The stain was 50mM Tris/HCl, pH 7·68; 8mM $MgCl_2$; 2mM glucose; 4mM ATP; 0·2mM NADP; 0·24mM nitroblue tetrazolium (NBT); 0·33mM phenazine methosulphate (PMS); and glucose 6-phosphate dehydrogenase (G6PD), 0·33 u/ml.

(b) 6-Phosphogluconate dehydrogenase (EC 1.1.1.44; 6-phospho-D-gluconate-$NADP^+$ oxidoreductase, decarboxylating). These were based on the method described above for hexokinase (Brewer & Sing, 1970). The gel and tank buffers were as for the previous enzyme, except that NADP was added to the cathode compartment to give a final concentration of 0·02mM to improve resolution and yield bands of higher intensity. The stain comprised 100mM Tris/HCl, pH 7·8; 10mM $MgCl_2$; 0·2mM NADP; 0·6mM 6-phosphogluconate; 0·33mM PMS; and 0·24mM NBT.

(c) Phosphoglucomutase (EC 2.7.5.1; α-D-glucose-1, 6-biphosphate: α-D-glucose-1-phosphate phosphotransferase). These were based on the method of Spencer *et al.* (1964). The reservoir buffer was 0·1M Tris; 0·1M maleic acid; 0·01M EDTA, disodium salt; and 0·01M $MgCl_2$; and the pH was adjusted to 7·6 with 5M NaOH. The gel buffer was a 10-fold dilution of the reservoir buffer. The stain comprised 100mM Tris/HCl, pH 7·3; 5mM $MgCl_2$; 0·2mM NADP; 5mM glucose 1-phosphate (containing 1% glucose 1,6-diphosphate); 0·24mM NBT; 0·33mM PMS; and G6PD, 0·33 u/ml.

(d) Lactate dehydrogenase (EC 1.1.1.27; L-lactate: $NAD^+$ oxidoreductase). These were based on the method of Dietz & Lubrano (1967). The reservoir buffer consisted of 5mM tris and 38mM glycine adjusted to pH 8·3. The stain was 50mM Tris/HCl, pH 8·0; 500mM sodium lactate; 2mM NAD; 0·24mM NBT; and 0·33mM PMS.

### RESULTS

### *Hexokinase*

Katzen & Schimke (1965) separated 4 zones of hexokinase activity from rat tissues, designated HKI–HKIV in order of increasing anodal mobility. HKI, II and III could be detected when the staining solution contained $5 \times 10^{-4}$M

glucose, whereas HKIV, found in liver, required 0·1M glucose concentration in the stain. HKIII, in contrast to HKI and II, was inhibited by glucose concentrations in the staining solution greater than about $10^{-2}$M, a result confirmed for human hexokinase isoenzymes by Rogers *et al.* (1975). In the present work no additional bands of activity were demonstrated in 11 normal and malignant specimens stained with solutions containing 0·1M glucose as opposed to 2mM glucose and, therefore, because of the danger of inhibiting HKIII if present, a concentration of 2mM glucose in the staining solution was routinely used for development of hexokinase isoenzymes.

Only samples with activity greater than 100 mu/ml proved sufficiently active for our detection system, of which 19 were from normal cervix epithelium, 19 were from malignant cervix and 8 were from malignant endometrium.

All showed a low mobility zone with a duplet structure presumably corresponding to the duplet zone of HKI described by Rogers *et al.* (1975). Many malignant samples, both of cervix and endometrium, showed another more mobile band corres-

FIG. 1.—Electrophoresis of hexokinase isoenzymes on starch gel. Origin (O) marks point of application of 5 malignant samples, all of which show duplet HKI isoenzyme, and Nos 1, 2 and 4 (from left) also demonstrate a zone of HKII activity towards the anode (+).

FIG. 2.—Electrophoresis of 6-phosphogluconate dehydrogenase on starch gel. Origin (O) marks application of 4 malignant samples, 3 of which (Nos 1, 2 and 4 from left) demonstrate the duplet band towards the anode (+).

ponding with HKII (Fig. 1). HKII was not detected in any of 19 samples of normal cervix, but was present in 6/19 samples of cervical cancer and 3/8 samples of endometrial cancer. Normal endometrium had insufficient activity to enable detection of hexokinase isoenzymes. No hexokinase isoenzymes other than I and II were observed in the material examined.

*6-Phosphogluconate dehydrogenase*

Of 25 normal cervix epithelium samples, all showed a single band of uniform mobility towards the anode. Malignant samples revealed a slightly slower band in addition to that of normal specimens (Fig. 2) in 22/32 samples. This additional band was seen in 19/25 samples of cervical cancer and in 3/7 samples of endometrial cancer. These specimens had been stored at $-20°C$ for up to 2 months. To check these findings, analyses were performed on a number of samples within 1 week of preparing the supernatant. When this was done, 10 normal and 23 malignant samples gave single bands, suggesting some structural change had taken place on storage, with reduction of net charge, increase in size, or both.

To accelerate the process of duplet formation, normal and malignant samples were incubated at 37°C, alone, and in the presence of 0·2mM NADP or 0·5mM mercaptoethanol before electrophoresis. After 24 h at 37°C, 3/5 malignant samples showed the appearance of a duplet, whereas 6 normal samples were unaltered. NADP and mercaptoethanol had no further effect. No correlation between haemoglobin content and the presence of double bands was found. It is unlikely that erythrocytes are the source of the second band.

*Phosphoglucomutase*

This enzyme is thought to be coded in 3 separate loci, designated $PGM_1$, $PGM_2$ and $PGM_3$ (Harris *et al.*, 1968). $PGM_1$ contains 3 common genetic variants noted by Spencer *et al.* (1964) which are transmitted by autosomal inheritance although, according to a more recent report (Bark *et al.*, 1976), further common phenotypes may

TABLE I.—*Distribution of $PGM_1$ phenotypes in normal and malignant uterine tissue samples*

| | Normal | | Cancer | |
|---|---|---|---|---|
| $PGM_1$ Phenotype | Cervix | Endometrium | Cervix | Endometrium |
| 1 | 15 | 9 | 17 | 33 |
| 2–1 | 16 | 3 | 11 | 4 |
| 2 | 1 | 1 | 2 | 1 |

FIG. 3.—Electrophoresis of phosphoglucomutase on starch gel, with diagrammatic representation of phenotypes at each of the 3 gene loci for this enzyme. The samples applied at the origin (O) demonstrate for the $PGM_1$ locus the following phenotypes 2, 1, 2–1, 2–1, and 1, respectively. The gene products at the other loci are not clearly resolved on this gel.

be detected by isoelectric focusing. Each homozygous phenotype presents after electrophoresis as an intense slow band and a more mobile band of lower intensity. The common heterozygotic phenotype of $PGM_1$ appears as 4 bands with mobilities and relative intensities of a mixture of the 2 homozygotes—that is, no hybridization occurs. Fig. 3 illustrates diagrammatically the appearance of the phenotypes and shows a typical electrophoretic separation. All 3 phenotypes occurred in normal and malignant cervix and endometrium, and their frequency is reported in Table I.

FIG. 4.—Electrophoresis of phosphoglucomutase on starch gel. Origin (O) marks application of 6 malignant samples. Prolongation of running and staining periods reveals 3 bands in first (from left) and 2 bands in the remainder at the $PGM_3$ locus. All samples show an "e" band and the first sample both "e" and "f" bands at the $PGM_2$ locus.

Harris *et al.* (1968) reported the distribution of PGM phenotypes in a random sample of the British population: 58% exhibited $PGM_1{}^1$, 36% showed $PGM_1{}^{2-1}$ and the remaining 6% had $PGM_1{}^2$. The combined normal samples of cervix and endometrium in the present study showed 53% $PGM_1{}^1$, 42% $PGM_1{}^{2-1}$ and 4% $PGM_1{}^2$, in good agreement with Harris *et al.* (1968). Malignant cervix samples did not vary significantly from these frequencies, but malignant endometrium showed a very high frequency of the $PGM_1{}^1$ phenotype ($\chi^2 = 11{\cdot}02$; $P < 0{\cdot}01$).

Genetic variants of the second locus, $PGM_2$, are rare. The usual form seen in erythrocytes consists of 3 bands decreasing in intensity with increasing mobility. In the uterine tissues studied only 2 bands were detected, the second (Band f) being absent in 13/18 malignant cervix samples, but present in all 18 normal cervix samples.

$PGM_3$ variants are common but could only be detected in samples with a high activity, as they accounted for a small proportion of total phosphoglucomutase. Even then, staining beyond the usual 3 h needed for optimal resolution of $PGM_1$ bands was necessary. Because of this and their high mobility, it was difficult to

TABLE II.—*Lactate dehydrogenase isoenzyme patterns in normal and malignant uterine tissue samples*

| | Normal | | Cancer | |
|---|---|---|---|---|
| Pattern | Cervix | Endometrium | Cervix | Endometrium |
| $LD_1$-shift | 4 | 5 | 10 | 9 |
| Symmetrical | 32 | 3 | 7 | 4 |
| $LD_5$-shift | 10 | 2 | 20 | 3 |

FIG. 5.—Electrophoresis of lactate dehydrogenase on polyacrylamide gel. Origin (O) marks beginning of running gel, with isoenzymes numbered $LD_5$ to $LD_1$ with increasing mobility towards the anode (+). The forward horizontal line shows the albumin–bromophenol-blue marker. Mixed normal and malignant samples were applied showing the following patterns: 1 and 3, $LD_1$ shift; 2 and 4, $LD_5$ shift; remainder symmetrical.

distinguish them while simultaneously resolving $PGM_1$ variants. $PGM_3$ was represented by 2–3 fast bands detected in 15/18 malignant cervix samples and 2/18 normal cervix samples (Fig. 4). Because of their faintness, we could not assess the frequency of variants at the $PGM_3$ locus.

*Lactate dehydrogenase*

Three patterns were distinguished in the samples studied: a symmetrical distribution about $LD_3$; a shift towards $LD_1$; and a shift towards $LD_5$. Table II demonstrates that, in general, normal cervix epithelium had a symmetrical pattern of LDH isoenzymes, whereas malignant cervix showed a shift towards either $LD_1$ or, more commonly, $LD_5$ (Fig. 5). The pattern of endometrial cancer was comparable to that of normal endometrium, both showing $LD_1$-dominance.

### DISCUSSION

*Hexokinase*

Hexokinase isoenzyme shifts involving an increase in HKII have been described in chemically-induced tumours of rat liver, mammary gland, and kidney (Shatton *et al.*, 1969), in experimental rat hepatomas (Sato *et al.*, 1969), in human hepatomas (Balinsky *et al.*, 1973; Hammond & Balinsky, 1978) and in all of 53 primary human tumours investigated (Kamel & Schwarzfischer, 1975).

The appearance of HKII in malignant uterine tissues is in agreement with the observations of Kikuchi *et al.* (1972) but, whereas they found HKII in all 10 samples of malignant cervix examined, only one third of the uterine tumours in our series showed HKII. On the other hand, Kikuchi *et al.* (1972) detected HKII in 3/10 samples of normal cervix, which may indicate contamination of their material with erythrocytes, which contain large amounts of HKII (Rogers *et al.*, 1975), or greater sensitivity of their detection system. In the present work, erythrocytes were removed by dissecting out blood clots and washing thoroughly with water. No mention of these precautions was made by Kikuchi *et al.* (1972). Haemoglobin estimations revealed very low levels in all but 4 of our samples demonstrating HKII, and the presence of the latter cannot be attributed to erythrocytes.

Since HKI and HKII differ in molecular weight, thermostability and appearance during foetal development, it has been suggested that they are determined by separate gene loci (Rogers *et al.*, 1975). Appearance of HKII in tumours would therefore represent reprogramming of protein synthesis. It has been suggested that this may be necessary for progression to neoplasia after the initiation event, and would permit a higher rate of glucose phosphorylation (Kamel & Schwarzfischer, 1975). Recent work supports the suggestion that increased HKII levels augment glucose utilization (Bernstein, 1977). Our failure to detect HKII in the majority of tumours precludes this change being an obligatory event in neoplasia. This conclusion is supported by a recent paper describing the presence of HKII in only 13/27 human glial tumours (Bennett *et al.*, 1978).

*6-Phosphogluconate dehydrogenase*

Latner (1967) reported a single band of PGDH in cervical carcinomas after starch-gel electrophoresis. The mobility was variable from tumour to tumour, but inequalities of mobility were abolished by adding excess NADP. He offered 3 explanations for this variability of the isoenzyme: (a) gene mutation; (b) ageing of cells in the population; and (c) variable NADP content of the tumours. Differences in electrophoretic mobility of 6-phosphogluconate dehydrogenase from malignant melanoma and normal skin have been described (Prasad *et al.*, 1974) Tumour. tissue occasionally showed a slower-moving band which was also present in muscle. The two bands differed in their binding capacity for NADP. During storage, many malignant samples developed a slower band of 6-phosphogluconate dehydrogenase after electrophoresis. Possible reactions resulting in loss of mobility are: loss of a cofactor, deamidation, desialysation, oxidation of sulphydryl groups, and conformational changes (Epstein and Schechter, 1968). Preincubation of normal and malignant samples with NADP or mercaptoethanol, however, evoked no change in mobility, whereas incubation at 37°C for 24 h resulted in duplet bands in 3/5 malignant samples. This reflects a difference in the microenvironment between the normal and the malignant state; perhaps the redox potential or the level of protein catabolism is the relevant factor.

*Phosphoglucomutase*

Assuming that the phenotypes observed for this enzyme in the uterus are characteristic of the individual genotype, the association between the $PGM_1{}^1$ phenotype and women with endometrial carcinoma may reflect association between the disease and some genetically determined aetiological factor. Women with $PGM_1{}^2$ may be less susceptible. A comparable situation has been demonstrated by Cassimos *et al.* (1973), who showed an association between glucose-6-phosphate dehydrogenase deficiency and a low incidence of cancer.

In the tissues studied, 2 instead of the 3 bands of $PGM_2$ seen in erythrocyte haemolysates were detected. This may represent genuine tissue variation or a feature of the electrophoretic technique. The second (Band f) was not detected in 13/18 malignant cervix samples, whereas all 18 normal cervix samples showed it. The most intense band (e), which was always present, may be the primary gene product and Band f may arise by subsequent modification; absence of Band f might suggest inactivity of the modifying agent in most cervical cancers.

Two or three fast bands representing the $PGM_3$ locus were detected in 15/18 malignant cervix samples and 2/18 normal samples. This difference in detection of $PGM_3$ bands presumably reflects an activity increase of enzymes of this locus due to malignant change. It is not due to higher total phosphoglucomutase activity of cancer samples *per se* since, as with other enzymes, the amount loaded on to the gel was quite constant. The significance of such an increase is uncertain. Although differences in specificity, affinity and molecular weight are known between the various PGM gene products (Harris *et al.*, 1968), the function of $PGM_3$ is enigmatic. $PGM_1$ is thought to be mainly involved with glucose-1-phosphate (G1P) as substrate, whereas $PGM_2$ uses ribose-1- phosphate (R1P) more efficiently. $PGM_3$ has a very high Km ($10^{-2}$M) for G1P and does not use R1P significantly.

*Lactate dehydrogenase*

Okabe *et al.* (1968) described a symmetrical pattern for the lactate dehydrogenase isoenzymes of normal cervix. Many workers (Turner, 1964; Sutcliffe & Emery, 1968; Latner *et al.*, 1966; Fottrell *et al.*, 1974) reported an increased $LD_5$ in samples of cervical carcinoma. Thus previous work is in agreement with the present findings, except for a study by Langvad & Pedersen (1969), who reported no significant increase in the ratio of $LD_4$ to $LD_2$ in 54 patients with carcinoma of the cervix. Their electrophoretic method did not allow visualization of $LD_5$, and there is difficulty in observing an $LD_2 \rightarrow LD_4$ shift compared with an $LD_1 \rightarrow LD_5$ shift. This finding of increased $LD_5$ content is in line with extensive studies of other human tumours (Goldman *et al.*, 1964; Nissen & Bohn, 1965) and is postulated to permit increased rates of glycolysis, since $LD_5$ is less susceptible to inhibition by pyruvate (Latner & Skillen, 1967).

The surprising observation of an $LD_1$ shift in about a quarter of the samples of carcinoma of the cervix has not been previously reported, although this phenomenon has been observed in other tumours (Henderson *et al.*, 1974; Prasad *et al.*, 1974). The proportion of H-subunits in human hepatomas seemed to be slightly greater than in normal or host liver (Hammond & Balinsky, 1978). Haemoglobin determinations revealed a somewhat high content in 3/19 tumour samples with $LD_1$ shift; haemoglobin concentrations in the same range were present in 7 tumour samples demonstrating $LD_5$ shift. It is therefore improbable that erythrocyte contamination can account for the observed instances of $LD_1$ shift in the malignant material.

Lactate dehydrogenase (LDH) isoenzyme patterns may change in tumours according to physiological stimuli rather than as a consequence of reprogramming of protein synthesis necessary for neoplastic growth. Oxygen tension, hormonal changes and virus interaction are reported to cause differential production of LDH subunits (Clark & Yochim, 1969; Prasad *et al.*, 1972). Perhaps the LDH isoenzyme pattern reflects the oxygen tension in a tumour. Superficial tumours directly accessible to oxygen may show an $LD_1$ shift, whereas deep tumours with poor vascularization may show an $LD_5$ shift. No such correlation was attempted in this work, but would be worth trying in future.

Significant differences in LDH isoenzyme patterns were not observed in endometrial carcinoma compared with normal endometrium. Fottrell *et al.* (1974) reported 70–76% M-subunits in 3 cases of carcinoma of human endometrium compared with 11% in normal endometrium samples during the proliferative phase of the menstrual cycle and 32% during the secretory phase. It is difficult to assess the significance of this work because of the small number of tumours examined, and because the variance of results in the control material was not indicated.

### CONCLUSION

Differences in the isoenzyme patterns for the 4 enzymes examined have been demonstrated between normal and malignant tissues of the human uterus. In the main, these changes are qualitative rather than absolute, and they did not show a significant degree of correlation in individual tumours or in the tumour population as a whole. No cancer-specific isoenzymic forms were elucidated. The closest to this objective was the failure of expression of the $PGM_3$ locus in normal cervix epithelium and the frequent occurrence of a duplet form of 6-phosphogluconate dehydrogenase in malignant cervix which was never seen in normal tissue. Further work is needed to elucidate the chemical basis for this phenomenon, and the genetic basis for the other differences, which may be due to changes in the cell population making up the biopsy sample. The relatively small number of samples did not encourage a detailed analysis of the relationship between the observed phenomena in cancers and the type of tumour, its sensitivity to therapy, its degree of invasiveness, and whether these phenomena have any prognostic significance. These goals are worthy of future study.

This work was generously supported by the Cancer Research Campaign. We are grateful to Dr C. B. Taylor for advice and criticism and to Mr David Millar who provided some of the normal samples used in this work.

### REFERENCES

BALINSKY, D., CAYANIS, E., GEDDES, E. W. & BERSOHN, I. (1973) Activities and isoenzyme patterns of some enzymes of glucose metabolism in human primary malignant hepatoma. *Cancer Res.*, **33**, 249.

BARK, J. E., HARRIS, M. J. & FIRTH, M. (1976) Typing of the common phosphoglucomutase variants using isoelectric focusing—a new interpretation of the phosphoglucomutase system. *J. Forensic Sci. Soc.*, **16**, 115.

BENNETT, M. J., TIMPERLEY, W. R., TAYLOR, C. B. & HILL, A. S. (1978) Isoenzymes of hexokinase in the developing, normal and neoplastic human brain. *Eur. J. Cancer*, **14**, 189.

BERNSTEIN, R. S. (1977) Control of rat adipose tissue hexokinase isoenzymes by sugars during *in vitro* incubation. *Proc. Soc. Exp. Biol. Med.*, **156**, 223.

BREWER, G. J. & SING, C. F. (1970) *An Introduction to Isozyme Techniques*. London: Academic Press. p. 77.

CASSIMOS, C., SKLAVUNU-TSURUKTOSGLU, D. C. & PANAJIOTIDIC, C. (1973) The incidence of G6PD disturbances in cancer patients. *Int. Res. Commun.* (March, 1950–1951.

CLARK, S. W. & YOCHIM, J. M. (1969) Lactic dehydrogenase activity, its isoenzyme distribution in the uterus of the rat and relationship to intrauterine oxygen tension. *Fed. Proc.*, **28**, 637.

DIETZ, A. A. & LUBRANO, T. (1967) Separation and quantitation of lactic dehydrogenase isoenzymes by disc electrophoresis. *Anal. Biochem.*, **20**, 246.

DRABKIN, D. L. & AUSTIN, J. H. (1932) Spectrophotometric studies: Spectrophotometric constants for common hemoglobin derivatives in human, dog and rabbit blood. *J. Biol. Chem.*, **98**, 719.

EPSTEIN, C. J. & SCHECHTER, A. N. (1968) An approach to the problem of conformational isoenzymes. *Ann. N.Y. Acad. Sci.*, **151**, 85.

FOTTRELL, P. F., SPELLMAN, C. M. & O'DWYER, E. M. (1974) Elevated levels of endometrial lactate dehydrogenase in hyperplasia and carcinoma of human endometrium. *Cancer Res.*, **34**, 979.

GOLDMAN, R. D., KAPLAN, N. O. & HALL, T. C. (1964) Lactic dehydrogenase in human neoplastic tissues. *Cancer Res.*, **24**, 389.

HAMMOND, K. D. & BALINSKY, D. (1978) Isozyme studies of several enzymes of carbohydrate metabolism in human adult and fetal tissues, tumor tissues, and cell cultures. *Cancer Res.*, **38**, 1323.

HARRIS, H., HOPKINSON, D. A., LUFFMAN, J. E. & RAPLEG, S. (1968) Electrophoretic variation in red cell enzymes. In *Hereditary Disorders of Erythrocyte Metabolism*. Ed. E. Beutler. New York: Grune & Stratton. p. 1.

HENDERSON, A. R., AHMAD, D. & MCKENZIE, D. (1974) Increased synthesis of lactate dehydrogenase "H" subunit by a malignant tumour. *Clin. Chem.*, **20**, 1466.

KAMEL, R. & SCHWARZFISCHER, F. (1975) Hexokinase isozymes in human neoplastic and fetal tissues: The existence of hexokinase II in malignant tumours and in placenta. *Humangenetik*, **30**, 181.

KATZEN, H. M. & SCHIMKE, R. T. (1965) Multiple forms of hexokinase in the rat: Tissue distribution, age dependency and properties. *Proc. Natl Acad. Sci.*, **54**, 1218.

KIKUCHI, Y., SATO, S. & SUGIMURA, T. (1972) Hexokinase isoenzyme patterns of human uterine tumours. *Cancer*, **30**, 444.

LANGVAD, E. & PEDERSEN, S. N. (1969) Lactate dehydrogenase patterns in the non-malignant and malignant uterine cervix. *Cancer*, **23**, 1171.

LATNER, A. L. (1967) Isoenzymes. *Adv. Clin. Chem.*, **9**, 69.

LATNER, A. L. & SKILLEN, A. W. (1967) *Isoenzymes in Biology and Medicine*. New York: Academic Press. p. 80.

LATNER, A. L., TURNER, D. M. & WAY, S. A. (1966) Enzymes and isoenzymes in pre-invasive carcinoma of the cervix. *Lancet*, ii, 814.

MARSHALL, M. J., GOLDBERG, D. M., NEAL, F. E. & MILLAR, D. R. (1978*a*) Enzymes of glucose metabolism in carcinoma of the cervix and endometrium of the human uterus. *Br. J. Cancer*, **37**, 990.

MARSHALL, M. J., GOLDBERG, D. M., NEAL, F. E. & MILLAR, D. R. (1978*b*) Properties of glycolytic and related enzymes of normal and malignant human uterine tissues studied to optimise assay conditions. *Enzyme*, **23**, 295.

NISSEN, N. I. & BOHN, L. (1965) Patterns of lactic acid dehydrogenase isoenzymes in normal and malignant human tissues. *Eur. J. Cancer*, **1**, 217.

OKABE, K., HAYAKAWA, T. & KOIKE, M. (1968) Purification and comparative properties of human lactate dehydrogenase isozymes from uterus, uterine myoma and cervical cancer. *Biochemistry*, **7**, 79.

PRASAD, R., PRASAD, N. & TEVETHIA, S. S. (1972) Expression of lactate and malate dehydrogenases in tumors induced by SV40 and 7,12-dimethylbenz(a)anthracene. *Science*, **178**, 70.

PRASAD, R., ROMSDAHL, M. M., SHAW, C. R., MUMFORD, D. M. & SMITH, J. L. JR, (1974) Isoenzyme variations in human malignant melanoma. *Cancer Res.*, **34**, 1435.

ROGERS, P. A., FISHER, R. A. & HARRIS, H. (1975) An electrophoretic study of the distribution and properties of human hexokinases. *Biochem. Genet.*, **13**, 857.

SATO, S., MATSUSHIMA, T. & SUGIMURA, T. (1969) Hexokinase isoenzyme patterns of experimental hepatomas of rats. *Cancer Res.*, **29**, 1437.

SHATTON, J. B., MORRIS, H. P. & WEINHOUSE, S. (1969) Kinetic, electrophoretic and chromatographic studies on glucose–ATP phosphotransferase in rat hepatomas. *Cancer Res.*, **29**, 1161.

SPENCER, N., HOPKINSON, D. A. & HARRIS, H. (1964) Phosphoglucomutase polymorphism in man. *Nature*, **204**, 742.

SUTCLIFFE, R. G. & EMERY, A. E. (1968) Lactate dehydrogenase isoenzymes in carcinoma of the cervix. *Manchester Med. Gaz.*, **47**, 22.